# PD-L1^+^ Regulatory B Cells from Rheumatoid Arthritis Patients Have Impaired Function in Suppressing IFN-ү and IL-21 Production

**DOI:** 10.3390/ijms26072998

**Published:** 2025-03-25

**Authors:** Mustafa Talib, Balázs Gyebrovszki, Anna Fodor, Anna Mészáros, Kata Balog Virág, Leila Gloria Barta, Bernadette Rojkovich, György Nagy, Gabriella Sármay

**Affiliations:** 1 Department of Immunology, Eötvös Loránd University, 1053 Budapest, Hungary; talibmustafa19@gmail.com (M.T.); gyebrovszki.balazs@gmail.com (B.G.); fodor.anna94@gmail.com (A.F.); annameszaros5@gmail.com (A.M.); leilagloria1997@gmail.com (L.G.B.); 2 Rheumatology-Rehabilitation Department, Buda Hospital of the Hospitaller Order of Saint John of God, 1027 Budapest, Hungary; rojkovich.b@gmail.com; 3 Department of Rheumatology and Immunology, Semmelweis University, 1023 Budapest, Hungary; nagy.gyorgy2@semmelweis.hu; 4 Heart and Vascular Center, Semmelweis University, 1122 Budapest, Hungary; 5 Department of Genetics, Cell- and Immunobiology, Semmelweis University, 1089 Budapest, Hungary

**Keywords:** helper T cells, IFNΥ, IL-21, PD1, PD-L1, regulatory B cells, rheumatoid arthritis

## Abstract

Rheumatoid arthritis (RA) is a systemic inflammatory autoimmune disease. The pathomechanism of RA depends on both B and T cells. Regulatory B cells (Breg) have been shown to suppress T-cell immune responses and play a key role in modulating autoimmune processes. We aimed to investigate the possibility of utilizing PD-L1^+^ Breg cells in downregulating the Th cells’ immune response in healthy individuals and RA patients. We hypothesized that the PD-1/PD-1L interaction plays a key role in this process, which may be defective in autoimmune diseases. We separated T and B cells from the peripheral blood of healthy volunteers and RA patients by magnetic cell sorting, and Th cells and Treg cells were isolated by fluorescence-activated cell sorting. The cytokine production by CD4^+^ Th cells was detected by intracellular flow cytometry. CpG and CD40L stimulations were applied to induce PD-L1^hi^ expressing Breg cells. We found that the frequency of PD-L1^hi^ cells is significantly lower in all B-cell subsets in RA compared to healthy controls. Functional analysis of induced PD-L1^+^ Breg cells in coculture with activated autologous Th cells has shown that healthy control samples containing higher levels of PD-L1^hi^ Breg cells significantly inhibit IFN-ү and IL-21 production by Th cells. In contrast, RA patients’ samples with lower levels of PD-L1^hi^ Breg cells failed to do so. Since the expression of PD-L1 on B cells can be modulated in vitro to induce Breg cell suppressive capacity, these data may provide new perspectives for future therapy for RA.

## 1. Introduction

Regulatory B cells (Bregs) represent a functionally distinct and phenotypically diverse subset of B cells responsible for inhibiting inflammatory responses by suppressing T cell activation and inducing regulatory T cell (Tregs) differentiation [1,2]. Although Bregs were primarily described as exerting their regulatory function via an IL-10-dependent pathway, IL-10-independent mechanisms, including transforming growth factor-β (TGF-β) and IL-35, are also implied. Additionally, Bregs can perform their function via cell–cell contact by using immune checkpoint molecules such as Programmed cell death ligand-1 (PD-L1) [3,4,5,6]. The lack of exclusive phenotypic markers for Bregs identification significantly impairs the comparability of studies, as nearly all B-cell subsets might have regulatory features depending on the context and various signals they receive [3]. In humans, Bregs were phenotyped as CD19^+^CD24^hi^CD38^hi^ transitional B cells [7,8,9], CD19^+^CD27^int^CD38^+^ plasmablasts [10], CD19^+^CD25^+^CD71^+^CD73^−^B regulatory 1 (Br1) cells [11], CD19^+^CD24^hi^CD27^+^B10 cells [12,13] CD19^+^CD25^+^ B cells [4], and Tim-1^+^ Bregs [14], all of which at least release either IL-10, TGF-β, or IL-35.

The importance of Bregs in inflammatory autoimmune diseases stems from their role in controlling inflammation. In multiple sclerosis (MS) patients, helminth infection increases the frequency of CD19^+^CD1d^hi^ IL-10^+^ Breg cells that suppress the proliferation and IFN-γ production by T cells, resulting in an improved clinical outcome compared to patients experiencing relapsing-remitting MS (RRMS) [15,16]. The functional and numerical deficiency of immature CD19^+^CD24^hi^CD38^hi^ B cells in systemic lupus erythematosus (SLE) results in their failure to differentiate into Bregs due to an elevated level of IFN-α, which favors plasmablast formation and consequently enhances autoantibody production and disease pathogenesis [16,17]. A similar phenomenon has been observed in rheumatoid arthritis (RA), where the frequency of IL-10^+^CD5^+^CD1d^hi^ B cells, IL-10^+^TIM1^+^ B cells, and CD24^hi^CD38^hi^ Bregs was reduced and accompanied by an inability to repress Th17 responses and convert CD4^+^ T cells into Tregs [9,18,19]. In our previous study, we also noticed a reduced number of CD27^+^IL-10^+^ B cells in RA patients, which were functionally impaired in suppressing CD4^+^ IFN-γ^+^ T cells in co-culture [20]. Accordingly, dysfunction of Bregs in RA may lead to persistent inflammation, which significantly worsens the prognosis of the disease.

Treatment of RA is mainly focused on reducing the inflammatory response. Recently, biologic therapies, such as rituximab (anti-CD20), have been used to deplete various B-cell subsets, significantly reducing the frequency and function of Bregs [21]. However, others have reported that the PD-L1^hi^ B-cell population in humans can suppress cTfh cell expansion and is refractory to B-cell depletion therapy [22]. Other therapeutic strategies used to treat RA are aimed at reducing inflammation, such as non-steroidal anti-inflammatory drugs (NSAIDs); disease-modifying anti-rheumatic drugs (DMARDs); new-generation biologic therapies designed to target-specific pathways, such as costimulatory signaling or the Janus kinase pathways; and combinations of these [23]. However, these treatments are associated with several adverse effects, and on the other hand, a significant proportion, around 30–40% of RA patients, are refractory and difficult to treat. Further research is, therefore, needed to develop more effective therapeutic strategies [24,25,26,27].

Loss of immune tolerance has been identified as a major factor in the development of autoimmune diseases, including RA. Breg and Treg cells play a critical role in restoring the balance of the immune system (see Supplementary Figure S1) [28]. Therefore, a better approach to treating RA would be to restore immune homeostasis by enhancing the function of regulatory cells that suppress B- and T-cell activation. However, due to their intermittent and multifunctional action, immunosuppressive cytokines, such as IL-10, may exacerbate RA pathogenesis by inducing B cell differentiation into IgM- and IgG-secreting plasmablasts, resulting in increased autoantibody titers [29]. Alternatively, a direct cell contact approach to modulate immune responses through checkpoint receptors, such as the PD-1/PD-L1 interaction—known to downregulate effective immune responses in the cancer microenvironment—may solve the problem. PD-1 is predominantly expressed on a variety of cells, including activated T cells, and the binding of PD-L1 would downregulate T cell activation [30]. Induction of PD-L1-expressing B cells (PD-L1^+^ Bregs) would likely improve the control of Th cell activation and restore immune tolerance. Accordingly, we aimed to evaluate the effect of in vitro-induced PD-L1^+^ Bregs on CD4^+^ Th-cell activity from RA patients and healthy controls (HCs) by monitoring CD4^+^ Th cell activation, proliferation, and cytokine production.

## 2. Results

### 2.1. B-Cell Subsets and PD-L1 Expression Are Differentially Distributed Among HCs and RA Patients

**Figure 1:**
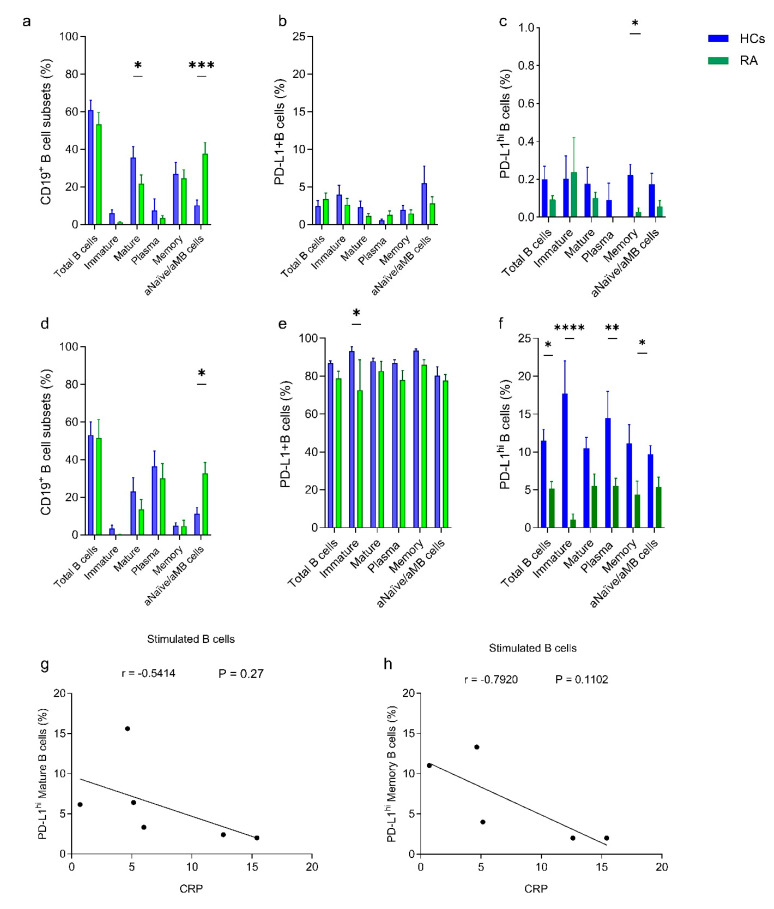
B-cell subsets and PD-L1 expression (**a**,**d**) showing the frequency of B-cell subsets before and after stimulation, respectively. B-cell subsets were gated as total B cells (CD19^+^ B cells), immature B cells (CD19^+^CD24^hi^CD38^hi^B cells), mature B cells (CD19^+^CD24^+^CD38^+^ B cells), plasmablast cells (CD19^+^CD24^−^CD38^+^ B cells), memory B cells (CD19^+^CD24^+^CD38^−^B cells) and activated naïve/memory B cells (CD19^+^CD24^−^CD38^−^B cells). Panel (**b**,**e**) represent the frequency of PD-L1^+^ B cells among the specified B-cell subsets before and after stimulation, respectively. Panel (**c**) depicts the frequency of PD-L1^hi^ Breg cells among PD-L1^+^ B cells from different B subsets before stimulation and (**f**) after stimulation. The results were calculated from healthy control (blue-filled bar chart, *n* = 8) and RA patients (green-filled bar chart, *n* = 8). The correlation between CRP and PD-L1^hi^ expression on mature and memory B cells was shown in (**g**,**h**), respectively, the analysis was performed for 5 to 6 patients for whom the required parameters were available. The B cells were stimulated for 96 h using CpG and sCD40L. The quantification is presented as mean ± S.E.M, using Ordinary Two-way ANOVA with uncorrected Fisher’s LSD. The significance was taken as * *p* < 0.5, ** *p* < 0.01, *** *p* < 0.001, **** *p* < 0.0001, aNaïve/aMB cells; activated naïve/activated memory B cells.

Breg cells serve a protective role in autoimmune disorders, and their frequency is inversely correlated with the disease severity of RA patients [18,19,31]. These cells are commonly known to downregulate T-cell activity by secreting anti-inflammatory cytokines such as IL-10 as well as repressing the inflammatory response in autoimmune disorders by expressing high levels of PD-L1 molecules [22,32]. In the current study, we evaluated the frequency of PD-L1 expressing B cells among the total CD19^+^ B cell population, CD19^+^CD24^hi^CD38^hi^ B cells (immature), CD19^+^CD24^int^CD38^int^ B cells (mature, naive), CD19^+^CD24^−^CD38^+^ B cells (plasmablasts), CD19^+^CD24^+^CD38^−^B cells (memory), and CD19^+^CD24^−^CD38^−^-activated naïve/activated memory B cells (aNaïve/aMB) [33] from RA patients and healthy individuals. First, we assessed the frequency of B-cell subsets before and after the stimulation with CpG+C40L; the gating strategy is shown at Supplementary Figure S2. The total CD19^+^ B cells were not significantly different between the two study groups (Figure 1a,d). However, healthy donors’ samples before stimulation show a higher frequency of mature, naive B cells. In contrast, the RA patients’ samples exhibit an abundant aNaïve/aMB-cell subset both before and after stimulation (Figure 1a,d). The frequency of PD-L1^+^ B cells, representing B cells with high and intermediate expressions of PD-L1, was not significant among the B-cell subset from healthy donors and RA patients before CpG stimulation. However, the frequencies of the PD-L1^+^ B cells were drastically increased within all B-cell subsets in both healthy and RA samples after the stimulation, and significantly fewer PD-L1^+^ B cells were observed among immature B cells from RA patients (Figure 1b,e).

PD-L1^hi^ Breg cells were reported to modulate Th-cell activity by IL-10-independent mechanism via interacting with PD-1 and were resistant to rituximab (anti-CD20) depletion [22]. Generally, we detected a very low level of PD-L1^hi^ Breg cells in both healthy and RA subjects with a higher frequency among memory B cells from healthy donors as compared to RA samples before stimulation (Figure 1c). After stimulation, PD-L1^hi^ Breg cells were significantly represented among different B-cell subsets from healthy donors (Figure 1f), while their frequencies were significantly lower among immature B cells, plasmablasts, and memory B cells from RA patients. Due to the scarcity of PD-L1^hi^ Breg cells, we investigated their regulatory effect as a part of the PD-L1^+^ B cell population without further purification.

We tested the correlation of PD-L1 expression with RA disease parameters, CRP, RF, aCCP, and DAS28. We found that PD-L1 expression on stimulated mature and memory B cells was negatively correlated with CRP (Figure 1g,h), and no correlation was observed with other RA disease parameters.

### 2.2. Expression of PD-1 on CD4^+^ Th and Treg Cells

**Figure 2:**
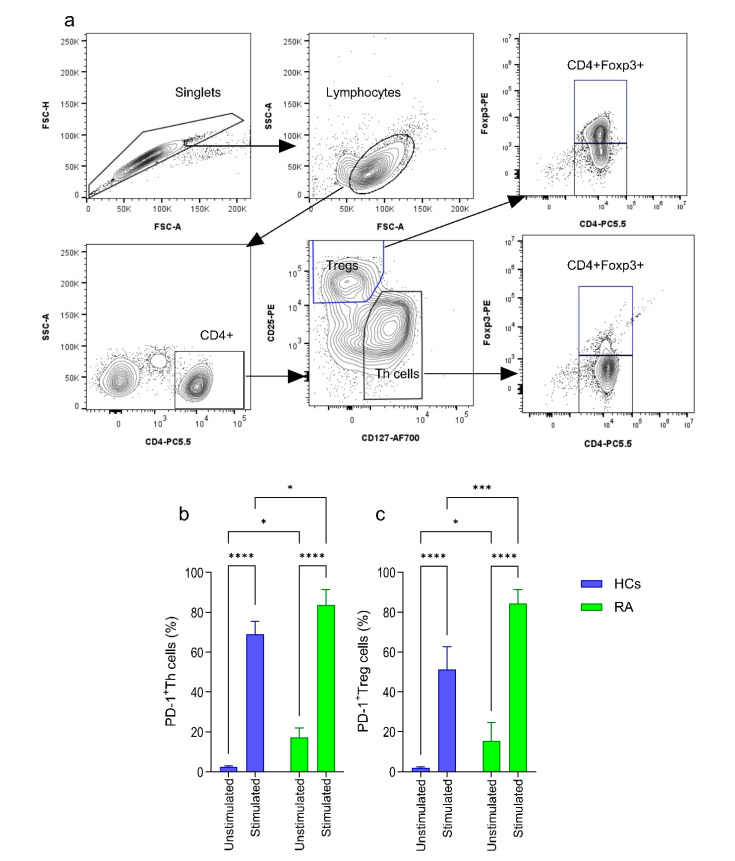
Assessment of PD-1 expression on Th/Treg cells. (**a**) Gating strategy of CD4^+^CD25l^o/−^CD127^+^Th and CD4^+^CD25^hi^CD127^lo/−^Treg cells sorted from peripheral blood and Foxp3 expression assessment among sorted Th and Treg cells. (**b**) Quantification of PD-1 expression on unstimulated and stimulated Th cells and (**c**) from Treg cells among HCs (*n* = 28) and RA patients (*n* = 19). All cell cultures were prepared by seeding 5 × 10^4^ Th/Treg cells in pre-coated plates with anti-CD3 and anti-CD28 monoclonal antibodies and stimulated for 96 h. The quantification is presented as mean ± S.E.M, using Ordinary Two-way ANOVA with uncorrected Fisher’s LSD. The significance was taken as * *p* < 0.5, *** *p* < 0.001, **** *p* < 0.0001.

PD-1 expression on T cells plays an important role in modulating their activity by mediating coinhibitory signals that suppress T cell activation when associated with their ligands PD-L1 or PD-L2 [29]. It has been reported that Th cells from RA patients express higher levels of PD-1 compared to healthy individuals [34], exposing them to the PD-L1 positive cells should downregulate their functional activity. We evaluated the expression of PD-1 on CD4^+^CD25^lo/−^CD127^+^ Th cells and CD4^+^CD25^hi^CD127^lo/−^Treg cells from RA patients and healthy controls. Th cells and Treg cells were sorted from MACS-enriched pan-T cells using fluorescent activating cell sorting and analyzed for the expression of the transcription factor Foxp3 (Figure 2a). As expected, unstimulated Th and Treg cells from RA patients expressed higher levels of PD-1 molecule compared to Th/Treg in healthy donor samples (Figure 2b,c). We induced PD-1 expression by stimulating Th and Treg cells using anti-CD3 + anti-CD28 coated plate, the frequency of PD-1^+^Th/Treg cells was robustly increased in both RA patients and healthy individual samples, but the highest frequency was observed in samples from RA patients (Figure 2b,c; Supplementary Figure S3). Accordingly, Th and Treg cells should be susceptible to the modulatory effect of PD-L1^+^ Breg cells.

### 2.3. Th and Treg Cell Proliferation Is Not Significantly Affected by PD-L1^+^ Breg Cells

**Figure 3:**
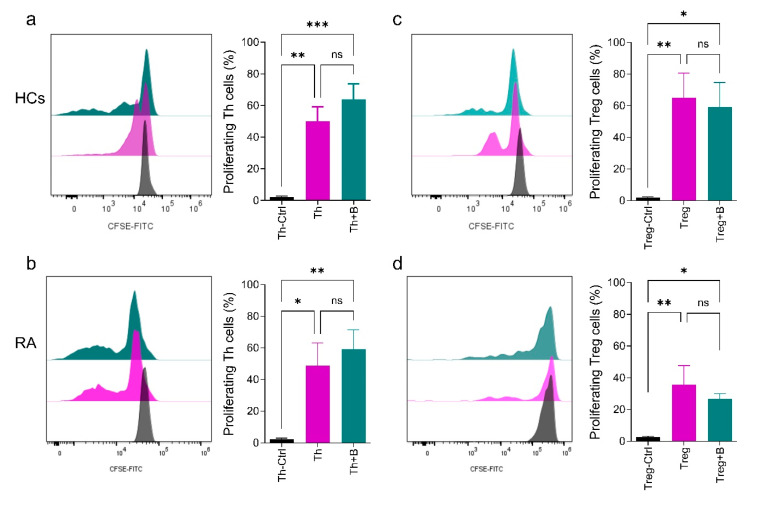
PD-L1^+^ Breg cell insignificantly affects the proliferation of Th (**a**,**b**) and Treg cells (**c**,**d**) from HCs and RA patients. The histograms to the left of each bar chart represent the proliferation pattern, and the bar chart depicts the quantification of the percentage of proliferating Th/Treg cells in unstimulated control, monoculture, or co-culture. At least (*n* = 9) and (*n* = 7) independent experiments from HCs and RA patients were used for quantification, respectively. Th and Treg cells were stimulated for 48 h by anti-CD3 and anti-CD28; for Treg cell stimulation, IL-2 was included in the cell culture. The co-cultures were prepared by adding pre-stimulated PD-L1^+^ Breg cells to the pre-stimulated Th/Treg cells, and all cultures were incubated for an additional 48 h before evaluation. The T cells and B cells were added into the culture at a 1:1 ratio. The data were evaluated using Ordinary One-way ANOVA with uncorrected Fisher’s LSD. The significance was taken as * *p* < 0.5, ** *p* < 0.01, *** *p* < 0.001. ns: not significant.

Previous works have demonstrated an impaired function of Breg cells in inhibiting CD4^+^ T cell proliferation and cytokine production in RA patients [9,20]. To test our hypothesis that the induction of PD-L1 expression in B cells from RA patients might enhance their regulatory effect, we evaluated the effect of PD-L1^+^ Breg cells on modulating the Th and Treg cells cell proliferation in an in vitro cell culture setting. The proliferation was assessed after Th or Treg cells were loaded with CFSE dye and stimulated in monoculture for 48 h before introducing the pre-stimulated PD-L1^+^ Breg cells for an additional 48 h. Following the dilution of CFSE in Th/Treg cells stimulated in monocultures and co-cultures (Th/Treg + B) as well as unstimulated Th/Treg cells (control), we observed that PD-L1^+^ Breg cells were unable to decrease Th cell proliferation in both RA and HC samples (Figure 3a,b, Supplementary Figure S4). However, the proliferation of Treg cells was insignificantly decreased in the samples from both donors (Figure 3c,d).

### 2.4. Functional Analysis of PD-L1^+^ Breg Cells by Comparing Cytokine Production

To further explore the induced PD-L1^+^ Breg cells’ role in manipulating the Th cells’ activity, we measured the production of IFN-γ, TNF-α, and IL-21, as these cytokines play a major role in triggering inflammation in RA and support antibody production by GC B cells as well as enhances local T cell activation in the synovium of RA patients [35].

**Figure 4:**
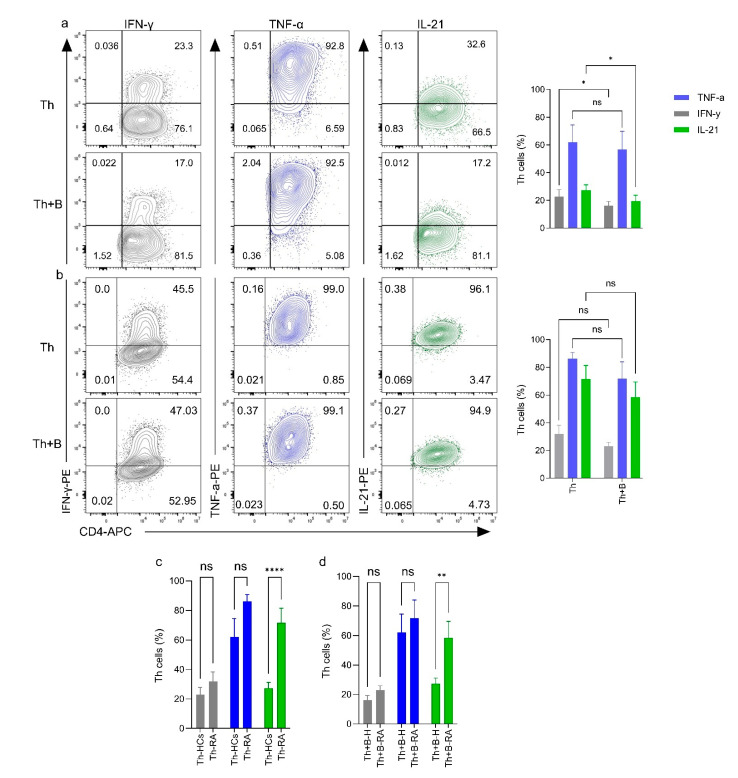
PD-L1^+^ Breg cells downregulate IFN-γ/IL21^+^ Th cells in HCs (**a**) but not in RA patients’ samples (**b**). On the left, a representative flow cytometry dot plot shows the percentage of IFN-γ/TNF-α/IL21 positive Th cells in activated Th cell monoculture (upper rows) and co-culture with PD-L1+ B cells (lower rows). On the right, the quantification of at least (*n* = 8) and (*n* = 7) independent experiments from HCs and RA patients, respectively. (**c**) a bar chart compares the production level of specific cytokines, IFN-γ (gray bar), TNF-α, (blue bar), or IL-21 (green bar), and a comparison of cytokine production between HCs and RA patients within similar culture conditions, in T cell monocultures (**c**), and in co-cultures of Th and PD-L1+ Bregs (**d**). The experiments were evaluated using Ordinary One-way ANOVA with uncorrected Fisher’s LSD. The significance was taken as * *p* < 0.5, ** *p* < 0.01, **** *p* < 0.0001. ns: not significant.

The production of these cytokines in the monoculture of activated Th cells and in their co-cultures with PD-L1^+^ Breg cells was monitored using intracellular staining protocol (Supplementary Figure S5). The frequency of TNF-α^+^ Th cells in monocultures from healthy donors was 62.0% ± 12.4%, and no significant reduction was detected by incorporating the autologous PD-L1^+^ Breg cells in the co-culture (mean ± s.e.m, 56.8% ± 12.9%, Figure 4a). Th cells from RA patients in monoculture produced a higher frequency of TNF-α^+^ (mean ± s.e.m, 86.2% ± 4.4% Figure 4c) compared to the corresponding monoculture from healthy donors. Similarly, the addition of PD-L1^+^ Breg cells did not significantly influence the frequency of TNF-α^+^ cells in the RA samples (mean ± s.e.m, 71.7% ± 12.2%, Figure 4b). The frequencies of IFN-ү^+^ Th cells in the monoculture were slightly different between healthy donors and RA patients (mean ± s.e.m, 22.9% ± 5.0% and 32.0% ± 6.3%, respectively, Figure 4a,b), and the addition of PD-L1^+^ Breg cells in co-cultures of healthy donors significantly reduced the frequency of IFN-ү producing Th cells (mean ± s.e.m, 16.1% ± 3.2%); this feature was not observed in RA patients’ samples (mean ± s.e.m, 23.1% ± 2.9%) (Figure 4a,b). Finally, IL-21^+^ Th cells were abundantly present in Th cells monocultures from RA patients compared to healthy controls (mean ± s.e.m, 71.5% ± 9.9% and 27.3% ± 3.8%, Figure 4c, respectively), and the autologous PD-L1^+^ Breg cells significantly reduced the frequency of IL-21^+^ Th cells in healthy donors’ samples but not in RA samples (mean ± s.e.m, 19.3% ± 4.4% and 58.5% ± 10.9%, Figure 4a,b, respectively). We further compared IFN-ү-, TNFα-, and IL-21-producing Th cells in T cell monocultures and in the co-cultures between the two groups; the frequency of IL-21-producing Th cells was significantly higher in both the monocultures and in the cocultures of RA patients’ samples (Figure 4c,d).

## 3. Discussion

Regulatory B cells are a subset of B cells that exert immunosuppressive activity mainly through the production of IL-10, TGF-β, and/or IL-35 or by highly expressing PD-L1 molecules [2,3,36]. As shown by Khan et al., the adoptive transfer of PD-L1^hi^ Breg cells into mice suppresses follicular T cell (Tfh) activity whereas the absence of these cells leads to increased Tfh responses [22]. The importance of Breg cells in rheumatoid arthritis stems from their role in manipulating the inflammatory immune response. We and others have previously shown that the frequency of IL-10^+^ Breg cells and their function are impaired in RA and negatively correlated with disease progression [20,37]. Disease management in RA patients primarily focuses on controlling the inflammatory response by administering synthetic or biological Disease Modifying Anti-Rheumatic Drugs (DMARDs) with target-specific immune system pathways. However, an alternative therapeutic approach to cure the disease would be to induce immune homeostasis by restoring regulatory mechanisms, such as modifying the functional activity of regulatory T and B cells. The present study investigates the efficacy of downregulating Th-cell activities by exploiting the expression of PD-1 and PD-L1 molecules on activated Th and Breg cells, respectively.

Breg function has been identified within multiple human B-cell subtypes [32]. However, in RA and several autoimmune disorders, B-cell subsets are abnormally distributed resulting in a skewed distribution of Breg cells [38,39,40,41,42]. In the current study, the aNaïve/aMB cells were the most prominent B-cell subset in the peripheral blood of RA patients, and their frequency was significantly higher in RA as compared to healthy controls. These CD19^+^CD38^−^CD24^−^ B-cell subset was previously detected at higher frequencies in the elderly population, and they were associated with TNF production, suggesting a possible link between this specific B-cell subset and the increased inflammatory status [42]. In vitro stimulation of B cells by CpG to generate PD-L1^+^ B (Bregs) cells resulted in the expansion of plasmablasts in both RA and healthy samples; however, aNaïve/aMB cells persisted as the most prominent B-cell subset in stimulated samples of RA patients. The abundance of aNaïve/aMB cells in both non-stimulated and stimulated RA samples suggests that the patients have a high risk of having uncontrolled inflammation. This is in concert with the high level of TNFα production even in the unstimulated RA samples (Figure 4).

Breg cells can modulate CD4^+^ T-cell activity by expressing high PD-L1 molecules [22,31], and they have been well documented among immature B cells and memory B cells [43,44]. However, for a comprehensive analysis, we have monitored the PD-L1^hi^ Breg cells among all our B-cell subsets including immature and mature B cells, plasmablasts, memory, and aNaïve/aMB cells. Although no significant difference in PD-L1 expression was observed, except in immature B cells of stimulated samples, we detected a significantly lower frequency of PD-L1^hi^ Breg cells among memory B cells of unstimulated RA samples. Furthermore, this low frequency was also observed in the immature B cell, plasmablast, and in the memory B-cell subset of CpG-stimulated RA samples compared to healthy controls. This is consistent with case–control reports from Luo et al. [45], who found that the proportions of CD19^+^CD24^hi^CD38^hi^ and CD19^+^CD24^hi^CD27^+^ Breg cells were significantly decreased in RA. We also described that PD-L1^hi^Breg cells were abundant among immature and memory B-cell subsets in CpG-stimulated samples from healthy controls, but their frequency was significantly decreased in RA samples, which may contribute to the uncontrolled inflammation in patients.

Th and Treg cells are key players in the pathogenesis of RA, as their functional activity significantly influences disease outcome [44]. We evaluated the activation status of Th and Treg cells from RA patients and healthy controls by measuring PD-1 expression. We found a significantly higher expression level of PD-1 molecule in RA compared to healthy controls both before and after CD3/CD28 stimulation indicating their active state [46]. High PD-1 expression should increase the possibility to interact with PD-L1^+^ Breg cells, which can downregulate Th cell functions, such as in the tumor microenvironment, where the interaction between PD-1/PD-L1 interaction leads to impaired antitumor immunity [9,22,47,48]. We, therefore, tested this hypothesis in co-cultures of CpG-stimulated PD-L1^+^ Breg cells and CD3/CD28-activated autologous Th cells. PD-L1^+^ Breg cells from both healthy and RA donors failed to inhibit Th cell proliferation in cocultures. This may be due to the heterogeneity of the CD4^+^CD25^lo/−^CD27^+^ Th cell subtype, as it may include different subsets at different developmental stages, and they may respond differently [49]. As PD-L1 interactions have been shown to modulate Treg-cell activity in mice, and Breg cells were supposed to promote differentiation of Tregs [50] thus we tested if Tregs proliferation is influenced by PD-1^+^ Breg cells in cocultures, but the difference in Treg proliferation in the presence and absence of Bregs was insignificant.

We further investigated the effects of induced PD-L1^+^ Breg cells on the IFN-γ, TNF-α, and IL-21 production by activating Th cells. PD-L1^+^ Breg cells from healthy individuals significantly downregulated both IFN-γ and IL-21 production in cocultures, whereas they did not reduce the production of any of the cytokines studied in RA patient samples, consistent with our previous study [46]. IL-21 production in activated Th cell monocultures from RA samples was approximately three times higher than in healthy controls and a similar difference was observed in cocultures with PD-L1^+^ Breg cells. These data suggest high functional activity of IL-21-producing Th cells in RA. As IL-21 is a key Tfh cytokine and plays a central role in supporting IgG production, targeting IL-21 could be an important factor in inhibiting autoantibody synthesis.

Taken together, these findings support the notion of impaired Breg cell function in RA patients [20,45] and shed light on the possibility of enhancing or restoring the regulatory function of Breg cells by inducing checkpoint molecule PD-L1 to downregulate the functional activity of Th cells in RA patients.

This research, however, is subject to some limitations. First, we had limited amounts of blood from the patients, which did not allow us to separate small subsets of cells, such as pTh, cTfh, and Bregs, for functional studies. Second, a larger number of patients in future studies would significantly improve the current results by minimizing the variance between donors, which would improve the power of the statistical analysis.

## 4. Methods

### 4.1. Patients and Controls

Peripheral blood samples were taken from 34 RA patients who did not receive biological therapy three months before sampling and met the American College of Rheumatology/European League Against Rheumatism Classification Criteria for rheumatoid arthritis [51] and 42 HCs. Patients and controls provided written informed consent. The blood samples were collected in heparinized VACUETTE^®^ TUBE (Greiner Bio-One, Mosonmagyaróvár, Hungary). The demographic and clinical characteristics of RA are summarized in Table 1. This study was conducted following the Declaration of Helsinki and ethically approved by the Scientific and Research Ethics Committee of the Health Science Council and the National Center for Public Health and Pharmacy (NCPHP) (42578-6-2018/EÜIG).

### 4.2. B and T Cell Isolation

Peripheral blood mononuclear cells (PBMCs) were isolated from 50 mL of fresh heparinized whole blood using density gradient centrifugation. The whole blood was diluted with 2 µM EDTA-PBS in a 1:1 ratio, and 30 mL was layered in Leucosep tubes (Greiner Bio-One, Mosonmagyaróvár, Hungary) containing 15 mL of PBMC Spin Medium (PluriSelect, Leipzig, Germany) and centrifuged for 15 min at 800× *g* at room temperature (RT) using a swing-out bucket rotor with soft stop brakes. The suspension above the porous barrier was transferred to a new 50 mL flacon tube and filled up to 50 mL with 2 µM EDTA-PBS, centrifuged at 300× *g* for 10 min, and the pelleted PBMCs were washed twice with 2 µM EDTA-PBS. Finally, the PBMCs were counted and stored at 4 °C overnight in 50 mL of complete RPMI-1640 medium (10% FCS, RPMI-1640 with L-glutamine, 1% streptomycin/penicillin (Sigma-Aldrich, St. Louis, MI, USA).

B and T cells were separated from PBMCs by magnetic sorting using negative selection with Pan B cell (Lot No. 5211003043, BioLegend, Merck Life Science Kft., an affiliate of Merck KGaA, Darmstadt, Germany) or Pan T cell (Lot No. 5220205924, BioLegend) isolation kits following the manufacturer’s instructions. The purity of B and T cells was evaluated by CytoFlex LX flow cytometry after staining with Brilliant Violet 421™ conjugated anti-human CD19 mAbs, BV421™ (Clone: HIB19, BioLegend), and phycoerythrin (PE) conjugated anti-human CD3 mAbs (Clone: UCHT1, BioLegend).

### 4.3. Flow Cytometry and Cell Sorting

CD4^+^ Th cells (CD4^+^CD127^+^CD25^low/−^Foxp3^−^) and Treg cells (CD4^+^CD127^low/−^CD25^hi^ Foxp3^+^) were sorted by FACSAria III cell sorter (Becton–Dickinson, Franklin Lakes, NJ, USA). For the identification of Treg cells, anti-CD4 PC5.5 (clone. RPA-T4), anti-CD25 PE (clone. BC96), anti-CD127 AF700 (clone. A019D5), and anti-mouse/rat/human Foxp3 AF488 (clone 150D) were used, all from BioLegend. The T cells were blocked for 20 min with 10% mouse serum-PBS (MS-PBS). The staining master mix from the antibody conjugates, except the Foxp3-specific antibody prepared in 5% FCS-PBS, was added to the blocked T cells and incubated for 30 min on ice. The stained T cells were washed twice with 10 mL 2% FCS-PBS, resuspended in 5% FCS-PBS, and filtered through a 70 µm strainer for sorting. Immediately after sorting, the Th and Treg cells were tested for Foxp3 expression using anti-mouse/rat/human Foxp3 Alexa Fluor 488 with the True-Nuclear™ Transcription Factor kits (BioLegend) according to manufacturer guidelines. Anti-CD25 PE and anti-CD279 AF488 (clone. NAT105, BioLegend) were used to evaluate the expression level of CD25 and PD-1. Intracellular cytokine production by Th cells was measured by using the BD cytofix/cytoperm^TM^ Plus Fixation/Permeabilization kits (cat: 555028, Becton–Dickinson) and the anti-IL-21 PE (clone: 3A3-N2), anti-IFNγ PE (clone: 4S.B3), or anti-TNFα PE (clone: Mab11) antibodies, all from BioLegend. The B cells were characterized by using anti-CD19 BV421™ (clone: HIB19, Biolegend), anti-CD24 FITC (clone: MI.5, BioLegend), anti-CD38 AF700 (clone: HIT2, ImmunoTools, Friesoythe, Germany), and anti-PD-L1 PE (clone:29E.2A3, BioLegend) antibodies.

### 4.4. B and Th Cell Activation

The isolated B cells were stimulated for 48 h in a RPMI-1640 medium with L-glutamine (BioWhittaker^®^, Lonza, Basel, Switzerland) supplemented with 10% fetal calf serum (FCS) and 1% streptomycin/penicillin (Sigma-Aldrich) at 5 × 10^4^ cells per well using round-bottom 96-well culture plates (cat: 83.392.500, SARSTEDT AG & Co.KG, Sarstedt, Germany) at 37 °C and 5% CO_2_ in the presence of 1 μg/mL phosphorothioated unmethylated CpG oligodeoxynucleotide (ODN-2006) 59-TCGTCGTTTTGTCGTTTTGTCGT-39; (Sigma-Aldrich) and 1 μg/mL rh-sCD40L (animal-free, Cat No.712904, Biolegend) to induce PD-L1^+^ Breg cells.

Th cells and Tregs, either stained with (5-(6) Carboxyfluorescein diacetate Succinimidyl ester “mixed isomer”), (CFSE) dye (Invitrogen TM, Waltham, MA, USA), or unstained, were stimulated for 48 h in flat-bottom 96-well culture plates (cat: 83.3924.500, SARSTEDT AG & Co.KG) pre-coated with 1 µg/mL Ultra-LEAFTM Purified anti-human CD3 (clone. UCHT1, Cat. 300438, BioLegend) and 1 ug/mL Ultra-LEAFTM Purified anti-human CD28 (clone. CD28.2, Cat: 302934, BioLegend). The Th cells and Tregs were added at 5 × 10^4^ cells/50 µL density, and 1 µg/mL rh-IL-2 (Cat:11340023, ImmunoTools, Friesoythe, Germany) was added to Tregs culture.

### 4.5. Proliferation Assay

The pre-stimulated CFSE-uploaded Th cells and Tregs were either assigned for monoculture or co-culture with the autologous PD-L1^+^ B (Bregs) cells, which were washed twice with complete RPMI 1640 to minimize any direct effect of preexisting cytokines from B cells culture supernatant, such as IL-10. The coculture was prepared at a one-to-one ratio and incubated for an additional 48 h to evaluate the immunomodulatory effect of induced Breg cells on Th cells and Treg cell proliferation. In parallel, unstimulated control from Th and Treg cell monocultures were prepared. The proliferation was evaluated by monitoring the CFSE dilution using flow cytometry after staining the cells with 7AAD to exclude the dead cells. The B cells were removed from the analysis by placing the gate on the CD4 positive region when applying anti-human CD4-APC (clone: EDU-2, ImmunoTools, Friesoythe, Germany) or CD19 negative gate when anti-human CD19 BV421™ was used.

### 4.6. Evaluation of Cytokine Production

We assessed intracellularly the production of TNF-α, IFN-γ, and IL-21 by Th cells derived from monoculture and in coculture with B cells. Th cell monoculture was prepared by seeding 5 × 10^4^ Th cells on a 96-well F-bottom plate, which was pre-coated with ant-CD3 and anti-CD28 monoclonal antibodies for up to 96 h, and the coculture was prepared by adding the induced Breg cells into the Th cell monoculture after 48 h stimulation. By the end of the stimulation period, the cells were stimulated for the last 5 h with 50 ng/mL Phorbol 12-myristate 13-acetate (PMA) (Sigma-Aldrich); 1 μg/mL Ionomycin (Sigma-Aldrich); and BD GolgiPlug™ protein transporter inhibitor containing Brefeldin A; 1:1000 (Becton–Dickinson). The cultures were harvested into 96-well U-bottom plates and washed twice with 200 µL staining buffer (FACS buffer) and further blocked with 10% mouse serum for 20 min on ice. Subsequently, the cells were washed twice before adding 100 µL of 2.5 µg/mL anti-human CD4-APC. The mixture was incubated in the dark for 30 min and washed twice with staining buffer before applying fixation and permeabilization steps using the BD Cytofix/cytopermTM Plus Fixation/Permeabilization kits (Becton–Dickinson) following the manufacturer's instruction. Finally, 2.5 µg/mL of anti-TNFα PE, anti-IFNγ PE, or anti-IL-21 PE was added for 30 min. After washing twice, the samples were resuspended into 200 µL staining buffer and measured by flow cytometry.

### 4.7. Statistical Analysis

The data were analyzed using an ordinary one- or two-way ANOVA, followed by an uncorrected Fisher’s LSD test using Prism 9 for Windows version 9.0.0 (121). The results were represented as mean ± SEM, and *p* < 0.05 was considered a significant difference.

## Figures and Tables

**Table 1:** Clinical and demographic data for RA patients.

	RA (*n* = 25)	Reference Value (RV)
Age (y; mean [range])	63.60 (43–84)	—
M/F (N)	4/30	—
Disease duration (y; mean [range])	15.00 (4.00–29.00)	—
Treatments:		
Methotrexate	6	—
Methotrexate + folic acid	1	—
Methotrexate + Medrol	1	—
Methotrexate + Hydroxychloroquine	2	—
Methotrexate + adalimumab-adaz	1	—
Methotrexate + folic acid + Medrol	1	—
Methotrexate + rituximab	2	—
Methotrexate + Medrol + tofacitinib	1	—
Certolizumab pegol + leflunomide	1	—
Leflunomide + Medrol	1	—
Leflunomide	1	—
Etanercept + Leflunomide	1	—
Adalimumab-adaz	2	—
Sulfasalazine + Methotrexate + Adalimumab	1	—
Adalimumab-adaz + hydroxychloroquine + Methotrexate + folic acid	1	—
Methotrexate + folic acid + sulfasalazine + adalimumab-adaz	1	—
Adalimumab + Methotrexate + folic acid + medrol+ hydroxychloroquine	1	—
CRP (mg/L; mean [range])	12.30 (0.58–53.00)	<8 mg/L
DAS28 [ESR-based] (mean [range])	4.00 (2.3–6.70.00)	
aCCP (IU/mL)		<20 IU/mL
aCCP −	*n* = 5	
aCCP + (mean [range])	2161.00 (25.00–32,000.00)	
RF (IU/mL)		<20 IU/mL
RF −	*n* = 8	
RF + (mean[range])	151.85 (13.00–961.00)	

Abbreviations: M/F: male/female, CRP: C-reactive proteins, DAS28: disease activity score using 28 joint counts, ESR: erythrocyte sedimentation rate, aCCP: anti-cyclic citrullinated peptide antibody, RF: rheumatoid factor, IU/mL: international units per milliliter.

## Data Availability

The data will be available upon request.

## Supplementary Materials

The following supporting information can be downloaded at https://www.mdpi.com/article/10.3390/ijms26072998/s1.

## Abbreviations

7AAD	7-AminoactinomycinD
ACPA	Anti-citrullinated protein antibody
CFSE	Carboxyfluorescein diacetate Succinimidyl ester “mixed isomer”
MACS	Magneticactivating cells sorting
MB	Memory B cells
PB	Plasma blast cells
PBMCs	Peripheral blood mononuclear cells
PC	Plasma B cells
PD-1	Programmed cell death protein 1
PMA	Phorbol 12-myristate 13-acetate
RF	Rheumatoid factor
Tfh	follicular T helper cells
Tph	peripheral T helper cells
