# Supplementary material for: PD-L1+ Regulatory B Cells from Rheumatoid Arthritis Patients Have Impaired Function in Suppressing IFN-ү and IL-21 Production
Source: Int J Mol Sci. 2025 Mar 25;26(7):2998. doi: 10.3390/ijms26072998 (PMC11988511; doi:10.3390/ijms26072998)
Supplement: Supplementary file 1 [file ijms-26-02998-s001.zip › ijms-3519511-supplementary.pdf]

International Journal of
Molecular Sciences

MDPI

Supplementary figures

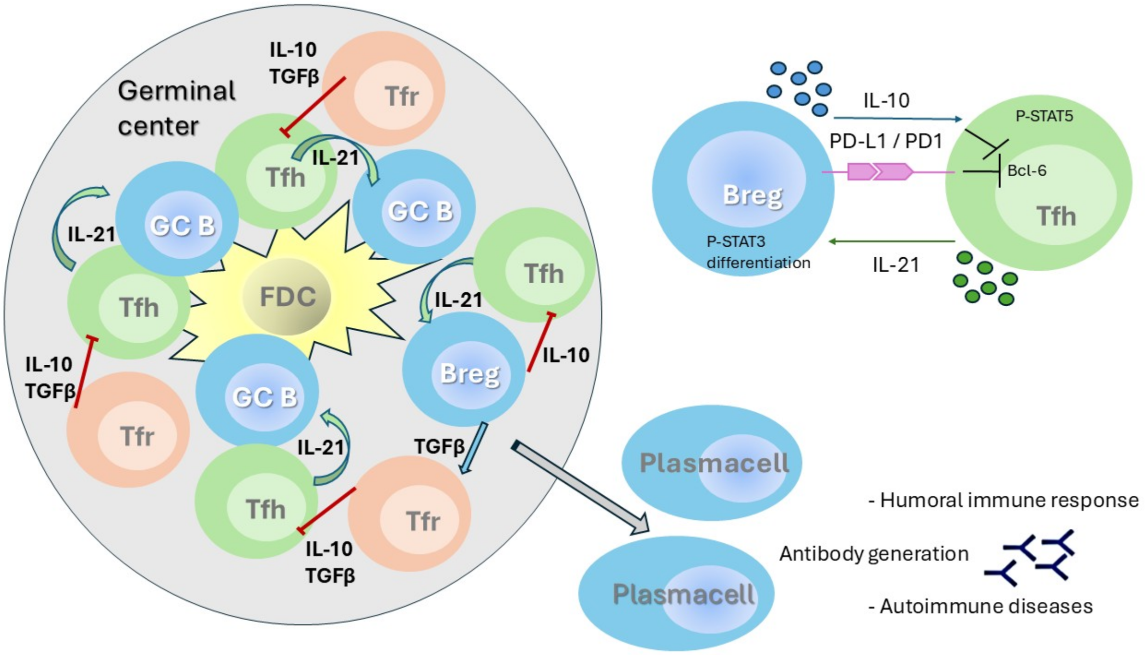

(**A**) (B)

Supplementary Figure S1. Regulation of the humoral immune response by the interactions between B cells, Tfh and Tfr cells in the germinal centre. (**A**) Tfh cells produce IL21, which supports B cell differentiation and antibody production. Tfr cells inhibit Tfh cell differentiation and function by secreting anti-inflammatory cytokines such as IL-10 and TGF-β, thereby inhibiting antibody production. (**B**) Breg cells express PD-L1 in an IL-21-dependent manner, and PD-L1hi Bregs can in turn downregulate Tfh function. Breg cells can also induce Tfr cell differentiation. An imbalance of these interactions may lead to autoimmunity [28].

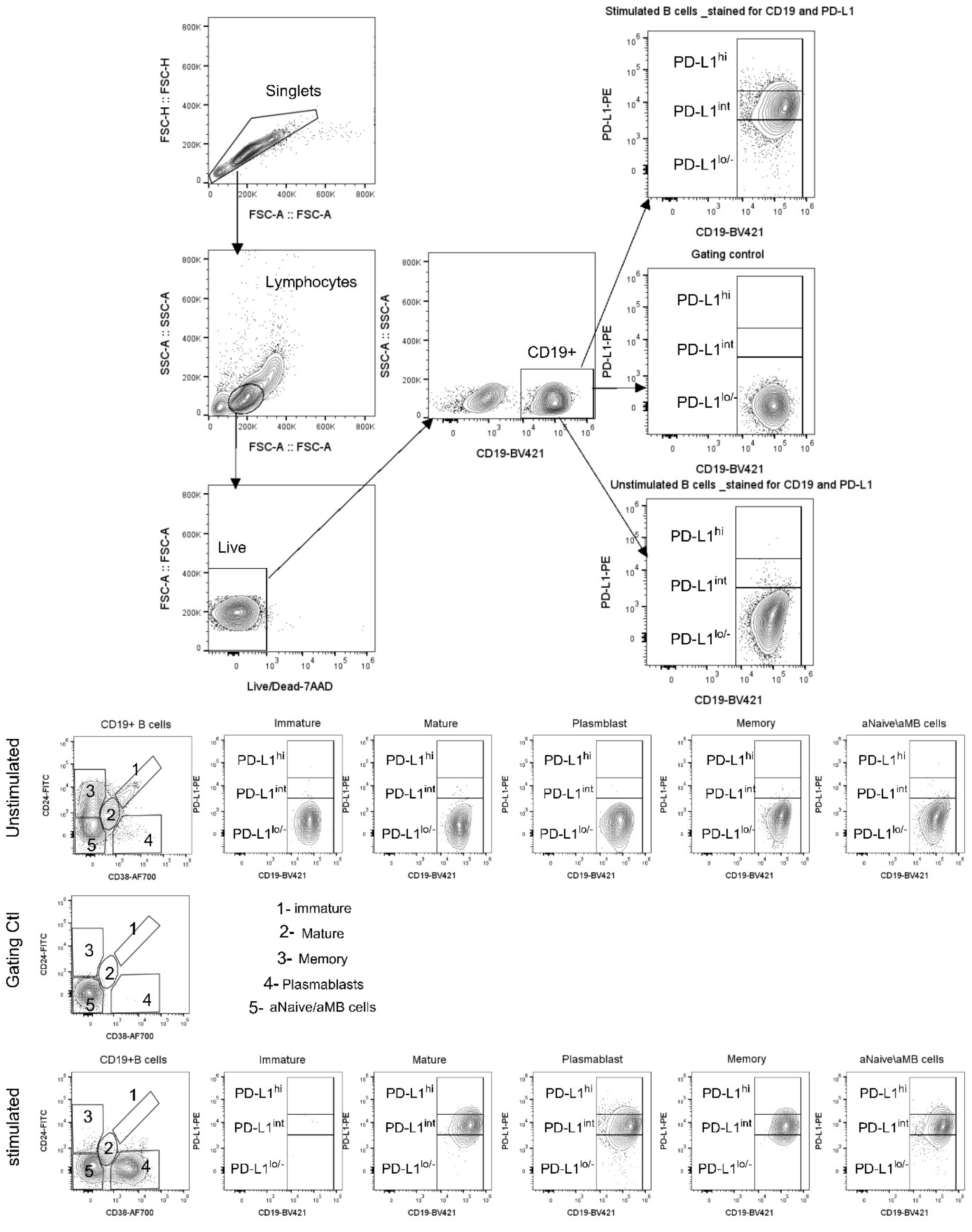

Supplementary Figure S2. Flow cytometry dot plot shows the gating strategy for B cell subsets using CD19, CD24, and CD38 markers, and PD-L1 expression (low/negative, intermediate, and high) by specific B cell subsets (Total B cells, Immature, Mature, Plasmablast, Memory, and activated naïve/activated memory B cells (aNaive/aMB cells) before and after stimulation.

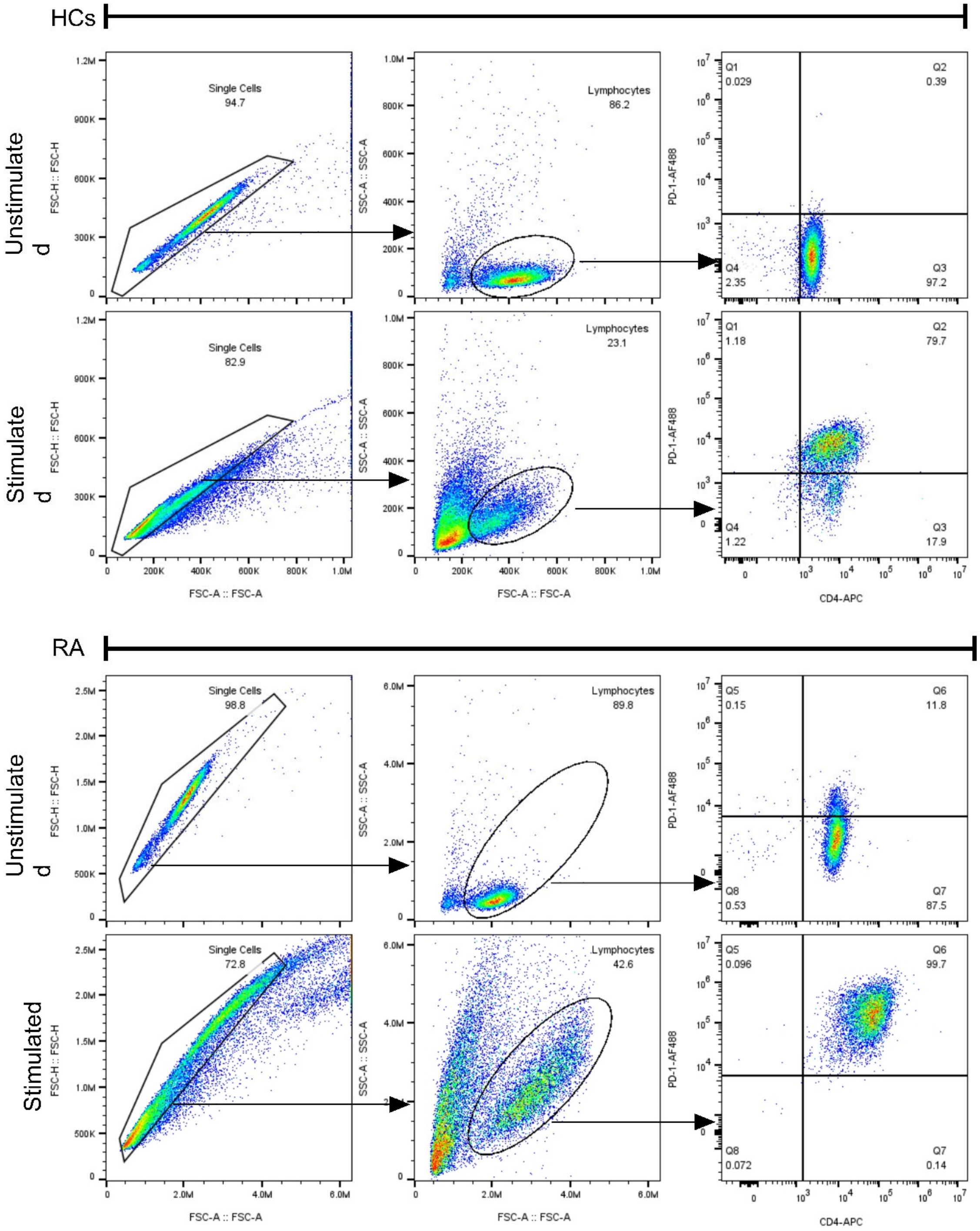

Supplementary Figure S3. The full gating strategy of PD-1 expression on Th cells before and after stimulation in HCs and RA patients.

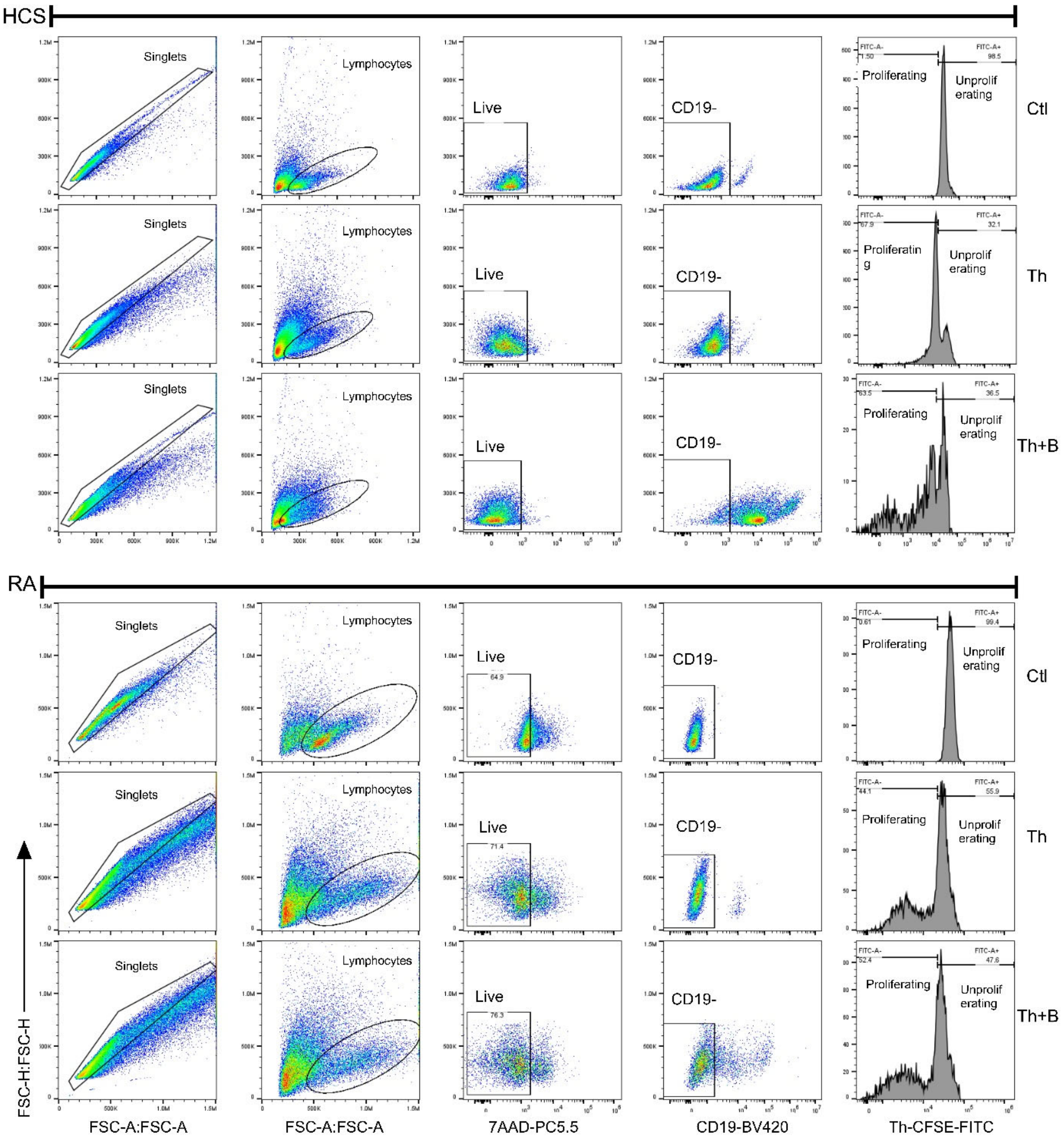

Supplementary Figure S4 Gating strategy for the proliferation assay. The co-culture (Th/Treg + PD-L1⁺B cells) was stained with anti-CD19-BV421 antibody conjugate to exclude the B cell from the analysis. The same gating strategy was applied for Treg cell proliferation evaluation.

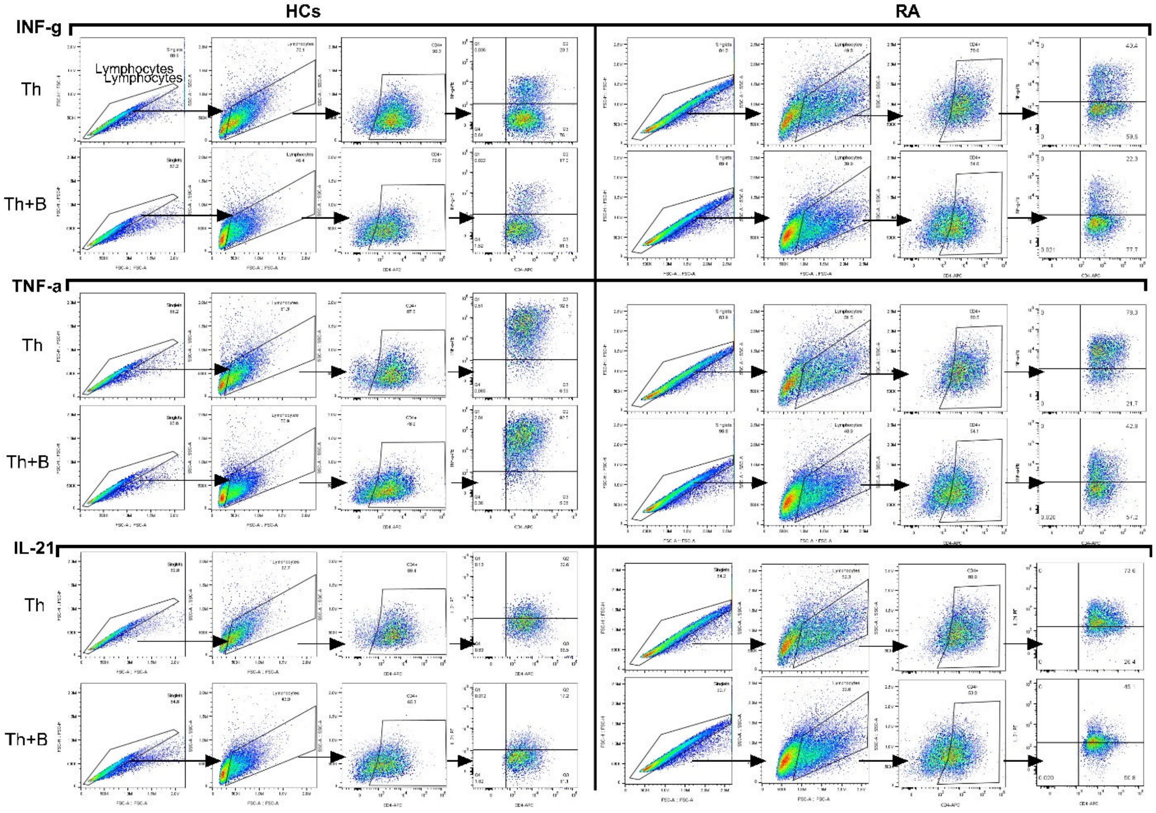

Supplementary Figure S5. A representative dot plot illustrates the expression level of pro-inflammatory cytokines, INF-γ, TNF-α, and IL-21 by Th cells in monoculture and co-culture among HCs and RA patients after 96h of stimulation.
